# Association of the MUTYH Gln324His (CAG/CAC) variant with cervical carcinoma and HR-HPV infection in a Chinese population

**DOI:** 10.1097/MD.0000000000015359

**Published:** 2019-04-26

**Authors:** Huaizeng Chen, Hanzhi Wang, Jia Liu, Qi Cheng, Xiaojing Chen, Feng Ye

**Affiliations:** a Women's Reproductive Health Key Laboratory of Zhejiang Province, Women's Hospital, School of Medicine, Zhejiang University; b Department of Obstetrics and Gynecology, Women's Hospital, School of Medicine, Zhejiang University, Hangzhou, Zhejiang, PR China.

**Keywords:** cervical squamous cell carcinoma, Chinese population, CIN III, HPV Infection, MUTYH Gln324His (CAG/CAC)

## Abstract

This study was performed to investigate the relationship between the MUTYH Gln324His (CAG/CAC) genotype and risk of cervical squamous cell carcinoma (CSCC) in a case-control setting. Mismatch amplification-polymerase chain reaction **(**MA-PCR) was applied to detect the polymorphism in 400 CSCC, 400 CIN III and 1200 control participants. The homozygous His324His (CAC/CAC) genotype of MUTYH was associated with significantly increased risk of CIN III (OR = 1.94) and CSCC (OR = 3.83). Increased risk of CIN III (OR = 1.34) and CSCC (OR = 1.97) was additionally observed with the heterozygous CAG/CAC genotype. Overall, individuals in both CAC/CAC and CAG/CAC genotype groups were at higher risk of cervical carcinoma (CINIII (OR = 1.46) and CSCC (OR = 2.34)). Within the HR-HPV infection-positive group, CAC/CAC and CAG/CAC genotypes were significantly enriched in relation to CIN III and CSCC. Moreover, we observed a positive correlation between the proportion of homozygous CAC/CAC MUTYH genotype and malignant prognostic factors of CSCC, such as cell differentiation grade and lymph node metastasis. These findings clearly highlight associations between the MUTYH Gln324His (CAG/CAC) polymorphism and susceptibility to CSCC, HR-HPV infection and specific prognostic factors, supporting the utility of this variant as an early indicator for patients at high risk of cervical carcinoma.

**Key points**

- For the first time, we investigated the genetic variant between cervical carcinoma and the base excision repair MUTYH Gln324His (CAG/CAC) polymorphism.
- High ratio and large scale normal controls made the results more reliable (1:3; 1200 normal controls).
- There was association between MUTYH Gln324His (CAG/CAC) polymorphism with the susceptibility of CSCC, HR-HPV infection, cell differentiation grade and lymph node metastasis, it may be used as an early warning indicator of high risk for cervical carcinoma, HR-HPV infection and prognostic factor.

## 1 Introduction

Cervical cancer (CC) is the third leading etiology of female mortality worldwide and associated with increasing rates of morbidity.^[1]^ High-risk human papillomavirus (HR-HPV) plays an important role in CC initiation and development.^[2,3]^

Globally, a combination of HR-HPV vaccination and HR-HPV-based screening strategies should theoretically control CC in any population in which a large coverage with both preventive options is ensured. However, accessibility to vaccination and the high cost of HR-HPV screening options in developing countries present significant barriers,^[2,3]^ leading to an intensive search for new primary or secondary CC prevention alternatives to complement existing programs. In this sense, research efforts have mainly been centered on genetic susceptibility factors to facilitate early detection of women at higher CC risk and generating evidence to identify women who should be directed without delay to more robust and stringent prevention programs, thereby achieving greater reduction of CC-induced mortality.^[4]^ Some previous studies have reported that the occurrence of tumor in a particular population is significantly associated with the genetic polymorphisms of certain genes, such as breast cancer and hepatocellular carcinoma.^[5–7]^ Epidemiological data further support the association of several genetic variants with risk of cervical cancer.^[8]^

Deficiencies of the DNA repair system are implicated in the development of cancer.^[9]^ The base excision repair (BER) pathway repairs the majority of endogenous DNA damage, including deamination, depurination, alkylation, and a plethora of oxidative damage.^[10]^ Several studies have reported potential associations of single nucleotide polymorphisms (SNP) of DNA repair genes with susceptibility to many solid cancers. Since SNPs of these genes may affect DNA repair activity, it is important to establish their roles in the molecular mechanisms underlying the tumorigenic process.^[11,12]^ MutY homologs (MUTYH, MYH) have been identified as key proteins in the BER pathway.^[13,14]^ Recent studies have demonstrated that bi-allelic germline mutations of the MUTYH gene lead to autosomal recessive colorectal adenomatous polyposis and very high colorectal cancer (CRC) risk in a Caucasian population.^[15,16]^ Przybylowska and co-workers reported that the MUTYH His324His (CAC/CAC) genotype is associated with increased CRC risk. Moreover, decreased efficiency of DNA repair was shown to be correlated with MUTYH Gln324His (CAG/CAC) genotype occurrence in CRC patients, suggesting a role in tumor pathogenesis.^[17,18]^ Another study by Singh et al^[19]^ suggested that MUTYH Gln324His (CAG/CAC) increases the risk of lung adenocarcinoma susceptibility.

To date, no reports have focused on the potential effects of the MUTYH Gln324His (CAG/CAC) SNP on cervical cancer. Accordingly, the present study was conducted to investigate the relationship between the MUTYH Gln324His genotype and risk of cervical squamous cell carcinoma (CSCC) in a case-control setting (including 400 CSCC, 400 precursor lesion CIN III and 1200 control participants). Determination of genetic associations can facilitate early diagnosis of individuals with increased risk of cancer and subsequent classification into different patient groups under different observation.

## 2 Materials and methods

### 2.1 Subjects

In total, 400 CSCC, 400 CIN III and 1200 normal control participants were recruited from Zhejiang Province, China. Diagnosis was confirmed by 2 pathologists. The normal control group included healthy women volunteers subjected to gynecologic examinations from June 2004 to December 2008. The criteria for recruitment of the normal control group included no positive cytological findings, no gynecological tumors, no endometriosis, no other cancer history and no immune disease. Overall, 201 cases with CSCC, 357 with CIN III and 609 control participants agreed to provide samples for the HR-HPV test. Our research was approved by the Medical Ethical Committee of Women's Hospital, School of Medicine, Zhejiang University (No.2004002). All patients signed informed consent for molecular research.

### 2.2 SNPs of MUTYH Gln324His (CAG/CAC, rs3219489) genotyping

Genomic DNA was extracted from peripheral blood according to the manufacturer's protocol (Sangon Bioengineering Co., Shanghai, China). All DNA samples were dissolved in water for experimental use.

SNPs of MUTYH Gln324His (CAG/CAC, rs3219489) were determined via modified polymerase chain reaction-mismatch amplification (MA-PCR) as described previously.^[20]^ The 2 forward primers of rs3219489 [G/C] were 5’-CAGCTCCCAACACTGGACTG-3’ for the G allele and 5’-CAGCTCCCAACACTGGACTC-3’ for the C allele, which differed only in the last base. The same reverse primer (5’-GGGCTCCCAGGTCACGGAC-3’) was used. The length of the amplified product was 343 bp (MUTYH gene DNA Reference Sequence: NG_008189.1).

PCR was performed in a 25 μl reaction volume containing 50 ng genomic DNA, 5.0 pmol each primer, 0.2 mM each deoxynucleoside triphosphate and 1.5 units of *Taq* DNA polymerase (TAKARA, Dalian, China) under the following conditions: initial denaturation at 94°C for 5 minutes, 35 cycles of 94°C for 30 seconds, 57°C for 30 seconds, and 72°C for 30 seconds, and a final elongation step of 72°C for 5 minutes. Amplified products were detected via 1.5% agarose gel electrophoresis and stained with ethidium bromide (EB). All samples were examined by 2 independent technicians and the results agreed for all masked duplicate sets. Data reproducibility was 100%.

### 2.3 High-Risk HPV (HR-HPV) detection

HR-HPV infection was determined using the Hybrid Capture II (HC II) assay (Digene Diagnostics Inc., Gaitherburg, MD, USA) conducted in keeping with the manufacturer's protocol. The assay includes RNA probes specific for HPV 16, 18, 31, 33, 35, 39, 45, 51, 52, 56, 58, 59, and 68.

### 2.4 Statistical analysis

To validate associations between the different genotypes and risk of cervical carcinoma, odds ratio (OR), 95% confidence intervals (CI) and *P*-values were obtained via binary logistic regression analysis. Stratified analyses of genotype frequencies by lifestyle habits and clinical pathological characteristics were evaluated with the Kruskal-Wallis H test with the control group set as the reference. All reported values are 2-tailed. The level of significance for differences was set at *P* ≤ .05. All statistical analyses were performed with SPSS for Windows software, version 18.0.

## 3 Results

### 3.1 Clinical characteristics of patients and normal controls

In the control, CIN III and carcinoma groups, 602/598, 258/ 142 and 160/240 individuals were ≤40 years />40 years of age, respectively. The carcinoma group contained significantly more individuals >40 years while the CIN III group had a significantly higher number of individuals < 40 years old, compared to the control. We observed no marked differences besides an increase in the proportion of individuals with number of parities >3 in the carcinoma and CIN III groups. The HR-HPV infection rate was 88.6% in the carcinoma and 86.8% in the CIN III group, compared to only 31.4% in the control group, clearly signifying higher infection rates in both CC groups (seen in Table 1). The data presented in Table 1 are quoted from our previously published paper. ^[21]^

**Table 1:**
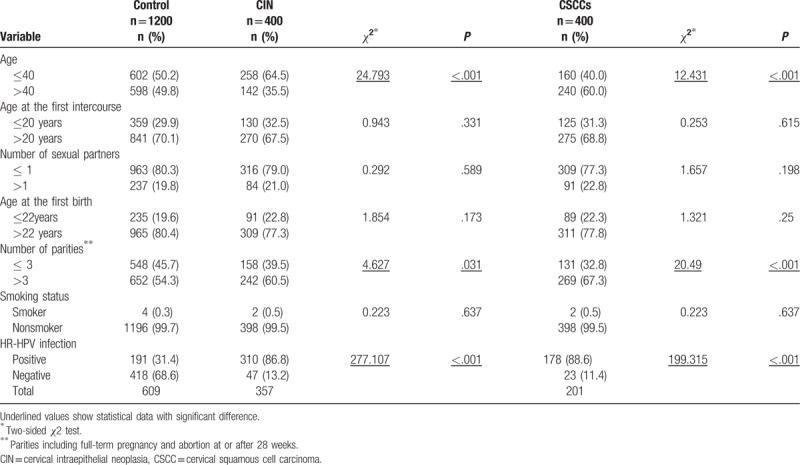
Frequency distribution of select characteristics by case control status.

### 3.2 Association between the MUTYH Gln324His (CAG/CAC) variant and risk of CSCC and CIN III

Table 2 depicts the genotypic and allelic frequencies of the MUTYH Gln324His (CAG/CAC) polymorphism. Genotype distributions were in Hardy-Weinberg equilibrium. Patients with the homozygous CAC/CAC genotype had significantly increased risk of CIN III (OR = 1.94) and CSCC (OR = 3.83). The heterozygous CAG/CAC variant of MUTYH was also associated with increased risk of CIN III (OR = 1.34) and CSCC (OR = 1.97)

**Table 2:**
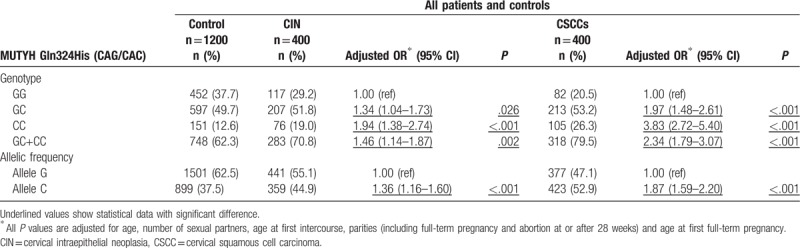
Association between MUTYH Gln324His (CAG/CAC) polymorphism and the risk of CIN and cervical carcinoma.

The G allele was the major form of the MUTYH Gln324His (CAG/CAC) polymorphism in the control group (62.5%) and the C allele in both CSCC (52.9%) and CIN III (44.9%) groups, compared with control (37.5%). Increased incidence of the C allele in correlation with CIN III and CSCC was confirmed (OR = 1.36 and OR = 1.87, respectively). Our data showed that individuals with CAC/CAC or CAG/CAC genotype at position 324 of MUTYH are at higher risk of CINIII (OR = 1.46) and CSCC (OR = 2.34).

As shown in Table 3, in the HR-HPV positive group, homozygous CAC/CAC genotype of MUTYH was correlated with significantly increased risk of CSCC (OR = 6.35) and CIN III (OR = 2.31). Patients with heterozygous CAG/CAC were also at increased risk of CSCC (OR = 2.41) and CIN III (OR = 1.51). Meanwhile, the proportion of C allele was significantly increased in the CIN III (46.3%, OR = 1.47) and CSCC (58.1%, OR = 2.38) groups, compared with control (36.9%). The CAC/CAC and CAG/CAC genotypes at MUTYH Gln324His (CAG/CAC) locus thus appear to be high risk factors for CSCC (OR = 3.18) and CIN III (OR = 1.66).

**Table 3:**
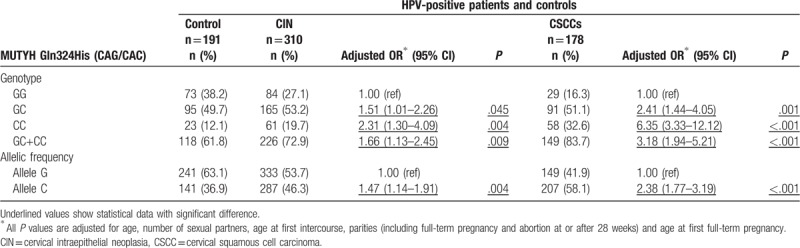
Association between MUTYH Gln324His (CAG/CAC) polymorphism and the risk of HPV-positive cervical carcinoma and CIN.

### 3.3 Association between MUTYH Gln324His (CAG/CAC) variants and sexual reproductive history in CSCC and CIN III

As shown in Table 4, participants were divided into 2 groups according to sexual and reproductive history and stratified analysis conducted in relation to the MUTYH Gln324His (CAG/CAC) genotype. Stratified analysis based on age, number of parities, and age at first parity revealed no correlation with MUTYH Gln324His (CAG/CAC) polymorphisms. However, we observed particularly high enrichment levels in relation to number of sexual partners between CIN III (*χ*^2^ = 6.358, *P* = .012) and CSCC (*χ*^2^ = 10.769, *P* = .001) and the age of first sexual intercourse between CIN III (*χ*^2^ = 10.286, *P* = .001) and CSCC (*χ*^2^ = 6.952, *P* = .008) groups.

**Table 4:**
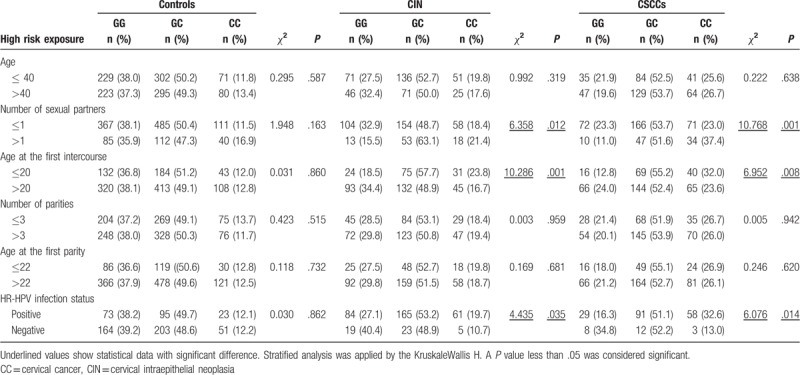
Association between MUTYH Gln324His (CAG/CAC) polymorphism and the risk for CIN and cervical carcinoma stratified by various environmental factors.

MUTYH Gln324His (CAG/CAC) polymorphisms in the HR-HPV-positive group were significantly enriched relative to the HR-HPV-negative group in CIN III (*χ*^2^ = 4.435, *P* = .035) and CSCC (*χ*^2^ = 6.076, *P* = .014) patients.

### 3.4 Association between MUTYH Gln324His (CAG/CAC) polymorphisms and clinicopathological characteristics in CSCC

Correlations of MUTYH Gln324His (CAG/CAC) polymorphisms with clinicopathological characteristics of CSCC are shown in Table 5.

**Table 5:**
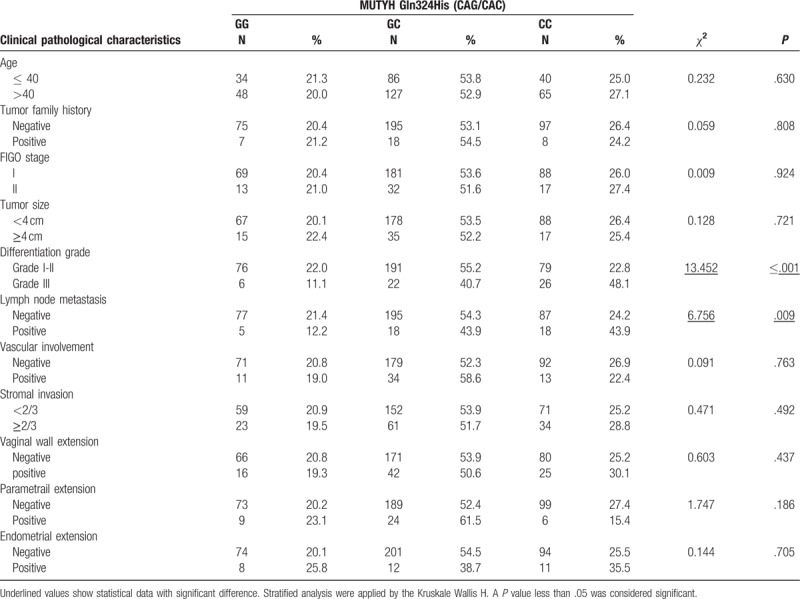
Association between MUTYH Gln324His (CAG/CAC) polymorphism and the risk for cervical carcinoma stratified by clinical pathological characteristics.

Stratified analysis based on age, tumor family history, FIGO stage, tumor size, vascular involvement, stromal invasion, vaginal wall extension, parametrial extension, and endometrial extension disclosed no correlation with MUTYH Gln324His (CAG/CAC) polymorphisms. However, we observed a particularly high level of enrichment following stratification by differentiation grade (*χ*^2^ = 13.452, *P* < .001) and lymph node metastasis (*χ*^2^ = 6.756, *P* = .009).

## 4 Discussion

Different biochemical processes involving oxygen lead to the formation of reactive toxic intermediates, producing reactive oxygen species (ROS) that attack genomic DNA. While life is oxygen-dependent, it also depends on complex mechanisms that can effectively detoxify potentially dangerous ROS and recover damaged DNA.^[10]^ Since oxidative damage of DNA triggers base mutations and long-term effects from irreversible mutations contribute to carcinogenesis, several studies to date have focused on DNA repair systems.^[22]^ The cellular antioxidant system provides the first line of defense against ROS-induced DNA damage. DNA-related mechanisms, including DNA repair, cell cycle arrest, and apoptosis, provide a further protection level. The DNA repair pathways efficiently antagonize oxidative damage. In the nucleus, oxidative DNA damage is repaired preferentially via base excision repair (BER), nucleotide excision repair (NER), mismatch repair (MMR), and recombination pathways.^[23]^ BER is responsible for repair of the majority of endogenous DNA damage, including plethoric oxidative damage, deamination, alkylation, and depurination reactions.^[10]^

MUTYH is a monofunctional DNA glycosylase that initiates the BER pathway by excising the adenine opposite 8OHG or 2- hydroxyadenine (2OHA), especially at the 2OHA: G mispair site. The MUTYH gene is composed of 16 exons.^[24,25]^ The N-terminal region containing the HhH element, followed by a Gly/Pro-rich loop and essential aspartate catalytical domain, interacts with the strand containing the substrate adenine residue, which is then entirely extruded from the DNA double helix and inserted into an extra helical pocket. This domain includes a [4Fe-4S] structural motif cluster, which is potentially involved in damage recognition as a transmitter of the oxidoreduction signal.^[26]^ The C-terminal region contacts the 8OHG-containing strand, thus leading to 8OHG recognition.^[27]^ Generally, DNA single nucleotide variants are associated with alterations in DNA glycosylase enzyme activity and base substitutions are common in the catalytic or substrate recognition domain. The enzymatic activities of 2 SNPs, Gln324His (rs3219489), and Val22Met (rs3219484), have been investigated. The Gln324 substitution occurs in the linker domain, which is not conserved among species, and may be involved in protein–protein interactions.^[28]^ Results on DNA glycosylase activity of the Gln324His mutant are paradoxical while no differences in the activities of the Val22Met variant located in the N-terminal region have been reported.^[29,30]^

Miyaishi et al analyzed the MUTYH Gln324His variants in 108 lung cancer cases and 121 normal controls in Japan. Their results showed that the MUTYH His/His homozygous genotype, compared with Gln/Gln homozygous genotype, is associated with increased risk for lung cancer (OR = 3.03 (1.31–7.00)) whereas no significant correlation was evident for the Gln/His genotype (OR = 1.35 (0.70–2.61)). The MUTYH His/His genotype was significantly associated with increased risk of both adenocarcinoma and squamous cell carcinoma (OR = 2.50 (0.95–6.62), OR = 3.20 (0.89–11.49), respectively, indicating an important role of this polymorphism in risk of lung cancer in the Japanese population. ^[31]^ A similar conclusion was drawn from the study of Singh et al on a population in India. Following DNA isolation from 326 lung cancer and 330 normal control cases and subsequent genotyping, the group found that single allelic carriers (Gln324His) for the MUTYH gene increased risk of overall lung cancer susceptibility.^[19]^

Przybylowska and co-workers had genotyped MUTYH polymorphisms in 182 CRC patients and 245 normal control subjects within a Polish population. The MUTYH Gln324His and His324His genotypes were found to be associated with increased CRC risk. Moreover, the XRCC1 Gln399Gln and MUTYH His 324His genotypes in patients were correlated with decreased efficiency of DNA damage repair capacity involved in risk of occurrence of CRC. The effects of these polymorphisms on DNA repair capacity strongly support a role in CRC pathogenesis.^[18]^ Similarly, Kasahara et al^[14]^ reported a significant relationship between the MUTYH Gln324His polymorphism and increased susceptibility to CRC in a Japanese population.

The above studies clearly suggest that the MUTYH Gln324His variant participates in the mechanisms of tumorigenesis. To date, however, no studies have analyzed the significance of this locus in cervical carcinoma. Accordingly, we have designed a correlation study to determine potential associations between MUTYH Gln324His and cervical carcinoma in a Chinese population.

Initiation of reversible changes in cervical cells involves various cellular abnormalities and ultimate progression to cervical carcinoma. Several well-defined phases of cervical carcinoma have been described, including precursor lesion cervical intraepithelial neoplasia (CIN) and malignant carcinoma.^[32]^ Data from the current study showed that the homozygous CAC/CAC genotype at position 324 of MUTYH is associated with significant risk of CIN III (OR = 1.94) and CSCC (OR = 3.83). Heterozygous MUTYH CAG/CAC variants at this position additionally led to increased risk of CIN III (OR = 1.34) and CSCC (OR = 1.97). Our results indicate that the MUTYH Gln324His (CAG/CAC) polymorphism participates not only in CSCC progression but also early molecular events of carcinogenesis.

We further analyzed enrichment of the MUTYH Gln324His (CAG/CAC) genotype in patients grouped by sexual and reproductive history, including number of sexual partners, age at first sexual intercourse, number of parities and age at first parity. A particularly high level of enrichment was evident upon stratification by number of sexual partners and age of first sexual intercourse between CIN III and CSCC groups. Numerous reports have confirmed that HR-HPV participates in the initiation and progression of cervical carcinoma.^[33]^ Within the HR-HPV infection-positive group, the CAC/CAC and CAG/CAC genotypes were more significantly enriched in CIN III and CSCC cases in our study, indicating that the MUTYH Gln324His (CAG/CAC) polymorphism is involved in initiation and progression of cervical carcinoma or precancerous lesions. Concurrently, we observed a positive correlation between the proportion of SNP and a number of malignant prognostic factors, such as cell differentiation grade and lymph node metastasis in patients with the homozygous CAC/CAC genotype, suggestive of poor prognosis. To our knowledge, the present study is the first to disclose an association between the MUTYH Gln324His (CAG/CAC) polymorphism and cervical carcinoma or CIN III.

Adenine removal (glycosylase) activity of MUTYH was demonstrated earlier by Ali and his co-workers, with rate constant (k_2_) values for MUTYH Gln324His and wild-type proteins of 4.288 ± 0.7831/minute and 4.507 ± 0.5812/minute, respectively. Moreover, only 64% adenine removal activity was detected with the mutant, compared to wild-type protein.^[29]^ Alterations in the spatial structure of the functional domain of the MUTYH (Gln324His) protein relative to its wild-type counterpart may contribute to decreased binding capacity and enzyme activity. 8-Oxo-G is generated by a hydroxyl radical whereas 2-OH-A is exclusively generated by oxidation of dATP in the nucleotide pool.^[25,34]^ The 2-OH-A level is enhanced in cancerous cells.^[35]^ Thus, in cervical carcinoma, enzyme activity of MUTYH Gln324His may be partially impaired.

Although our findings are very clear and reliable, our research is still limited to the SNP locus and disease-related research. We have not carried out the detection of MUTYH protein expression changes and functional differences, current results can not draw the conclusion that protein function may change. At the same time, because we only study single SNP locus of MUTYH gene, we can consider more loci detection and linkage disequilibrium analysis in future research, so as to obtain more comprehensive conclusions.

Briefly, our results suggest that MUTYH Gin324His (CAG/CAC) heterozygotes express defective proteins with impaired function, leading to consequent effects on genomic stability. Based on the collective findings, we conclude that the MUTYH Gln324His (CAG/CAC) genetic variant could serve as an early indicator of high risk of cervical carcinoma, HR-HPV infection and a prognostic factor. Future studies will focus on extensive characterization of the interactions of wild-type and MUTYH (Gln324His) proteins in cervical carcinogenesis.

## Acknowledgments

The authors are grateful to Drs. Caiyun Zhou and Minhua Yu for their assistance in recruiting the subjects. We thank International Science Editing (http://www.internationalscienceediting.com) for editing this manuscript.

## Author contributions

Conceived and designed the experiments: FY HC. Performed the experiments: FY HW QC JL XC. Analyzed the data: FY HC. Contributed reagents/materials/analysis tools: FY HW QC. Wrote the paper: FY HC.

**Conceptualization:** Huaizeng Chen, Feng Ye.

**Data curation:** Jia Liu.

**Funding acquisition:** Huaizeng Chen, Feng Ye.

**Investigation:** Huaizeng Chen, Jia Liu, Feng Ye.

**Methodology:** Hanzhi Wang, Qi Cheng, Xiaojing Chen.

**Validation:** Hanzhi Wang.

**Writing – original draft:** Huaizeng Chen.

**Writing – review & editing:** Feng Ye.
